# Stress and Its Effects on Medical Students: A Cross-sectional Study at a College of Medicine in Saudi Arabia

**DOI:** 10.3329/jhpn.v29i5.8906

**Published:** 2011-10

**Authors:** Hamza M. Abdulghani, Abdulaziz A. AlKanhal, Ebrahim S. Mahmoud, Gominda G. Ponnamperuma, Eiad A. Alfaris

**Affiliations:** ^1^Department of Medical Education, KSU Medical Education Chair for Research and Development, College of Medicine, King Saud University, PO Box 230155, Riyadh 11321, Saudi Arabia; ^2^Department of Surgery, College of Medicine, King Saud University, Riyadh, Saudi Arabia; ^3^Department of Medicine, Security Force Hospital, Riyadh, Saudi Arabia; ^4^Medical Education Development and Research Centre, Faculty of Medicine, University of Colombo, Colombo, Sri Lanka; ^5^Department of Family and Community Medicine, KSU Medical Education Chair for Research and Development, College of Medicine, King Saud University, Riyadh, Saudi Arabia

**Keywords:** Academic achievements, Cross-sectional studies, Impact studies, Medical education, Stress, Saudi Arabia

## Abstract

Medical education is perceived as being stressful, and a high level of stress may have a negative effect on cognitive functioning and learning of students in a medical school. This cross-sectional study was conducted to determine the prevalence of stress among medical students and to observe an association between the levels of stress and their academic performance, including the sources of their stress. All the medical students from year one to year five levels from the College of Medicine, King Saud University, were enrolled in the study. The study was conducted using Kessler10 psychological distress (K10) inventory, which measures the level of stress according to none, mild, moderate, and severe categories. The prevalence of stress was measured and compared with the five study variables, such as gender, academic year, academic grades, regularity to course attendance, and perceived physical problems. The response rate among the study subjects was 87% (n=892). The total prevalence of stress was 63%, and the prevalence of severe stress was 25%. The prevalence of stress was higher (p<0.5) among females (75.7%) than among males (57%) (odds ratio=2.3, χ^2^=27.2, p<0.0001). The stress significantly decreased as the year of study increased, except for the final year. The study variables, including being female (p<0.0001), year of study (p<0.001), and presence of perceived physical problems (p<0.0001), were found as independent significant risk factors for the outcome variable**s** of stress. Students' grade point average (academic score) or regularity to attend classes was not significantly associated with the stress level. The prevalence of stress was higher during the initial three years of study and among the female students. Physical problems are associated with high stress levels. Preventive mental health services, therefore, could be made an integral part of routine clinical services for medical students, especially in the initial academic years, to prevent such occurrence.

## INTRODUCTION

The medical school curriculum of the College of Medicine, King Saud University, Riyadh, Saudi Arabia, has been developed to graduate competent and professional physicians to serve their community efficiently. Medical education is perceived as being stressful, as it is characterized by many psychological changes in students.

Studies have shown that medical students experience a high level of stress during their undergraduate course (1-5). High level of stress may have a negative effect on cognitive functioning and learning of students in the medical school (6). Results of studies suggest that mental health worsens after students begin medical school and remain poor throughout the training (1). The majority of studies on stress in medical education focus on the documentation of stress and information on the correlates of stress (4,7).

In many medical schools, the environment itself is an all prevailing pressure situation, providing an authoritarian and rigid system, one that encourages competition rather than cooperation between learners (5). It is not just the undergraduate study period which brings stress but it may continue during the internship, postgraduate study period, and later into physician's practical life (8-10). The stress may also reach burnout levels (11).

The estimated prevalence of emotional disturbance found in different studies on medical students was higher than that in the general population. In three British universities, the prevalence of stress was 31.2% (12), and it was 41.9% in a Malaysian medical school (13) and 61.4% in a Thai medical school (14). Stress in medical school is likely to predict later mental health problems but students seldom seek help for their problems (15). In a Swedish study, the prevalence of depressive symptoms among medical students was 12.9%, and 2.7% of students had made suicidal attempts (6). It is important for medical educators to know the prevalence, causes, and levels of stress among students, which not only affect their health but also their academic achievement**s** at different points of time of their study period.

In Saudi Arabia, local epidemiological data about psychological morbidity among medical undergraduate students are scarce. Results of two recent studies from Egypt and Saudi Arabia suggest high rates of anxiety and depression among medical students (16,17). An extensive electronic Internet-based search failed to locate any study which shows an association between stress and academic achievement in undergraduate medical students in Saudi Arabia. The present study was, therefore, carried out to determine the prevalence of self-perceived stress among medical students and to observe any possible association between the levels of stress and (a) gender, (b) academic year, (c) academic grades, (d) regularity of course attendance, and (e) presence of perceived physical problems.

## MATERIALS AND METHODS

### Instrument

A wide range of different measures have been used for addressing psychological distress and depressive symptomatology among medical students, such as Beck's Depression Inventory (13), General Health Questionnaire (GHQ) (11), and other common and less common instruments (6,18).

We used the Kessler10 Psychological Distress instrument (K10) developed by Kessler and colleagues (19). This instrument has been used widely in population-based epidemiological studies to measure current (1-month) distress and was translated in different languages, including Arabic, to measure the level of stress and severity associated with psychological symptoms in population surveys. The World Mental Health Survey of the World Health Organization used it as a clinical outcome measure (20-23). The K10 consists of 10 questions in the form of “how often in the past month did you feel …” and offers specific symptoms, such as ‘tired out for no good reason’, ‘nervous’, and ‘sad or depressed’. The five possible responses for each question range from ‘none of the time’ to ‘all of the time’ and were scored from 1 to 5 respectively. All the questions were collated to obtain a total score. The total score was interpreted as follows: a score of less than 20 was considered not to represent stress of any level while a score of 20-24 represented mild stress, 25-29 represented moderate stress, and 30-50 represented severe stress (21). The questionnaire had also additional questions relating to academic achievement, source**s** of stress, and any perceived medical illness.

The K10 questionnaire was observed to have good psychometric properties with a Cronbach's alpha of 0.89 [95% confidence interval (CI) 0.88-0.90].

### Study subjects

All the male and female medical students in the five academic years of the College of Medicine, King Saud University, were invited to complete the bilingual (Arabic and English) version of the K10 self-administered, anonymous questionnaire during the 2007-2008 academic year.

### Collection of data

Completed questionnaires were collected two months before the examination period so that the actual examination stress would not affect the responses of the students. Responses to additional questions relating to academic achievement, sources of stress, medical illness in the past four weeks, and how many days a student was not able to work were also collected. The students were allowed to respond in their own time and privacy. The participation was entirely voluntary.

### Analysis of data

Data were entered in Microsoft Excel and analyzed using the SPSS software (version 16.0). The outcome variable—stress—was categorized dichotomously as stress (no/yes). The three levels (mild, moderate, and severe) of stress were put into one category and titled as ‘presence of stress’. Descriptive statistics (mean, standard deviation, and percentages) were used for summarizing the study and outcome variables. Pearson's chi-square test for trend and odds ratios were used for observing and quantifying the association between a categorical outcome (i.e. the stress level) and different study variables. Student's *t*-test for independent samples was used for comparing the mean values of continuous study variables in relation to stress levels. Multiple logistic regression was used for identifying the independent risk factors of stress. The 95% confidence intervals were calculated for both odds ratios (unadjusted and adjusted). A p value of <0.05 was considered significant.

### Ethical aspects

All students who participated in the study were informed about the objectives of the study, and the information about the instrument was explained by well-trained students who acted as research assistants. Approval for conducting the study was obtained from the research ethical committee of the College of Medicine, King Saud University.

## RESULTS

In total, 775 (87%) of approximately 892 students completed the questionnaire Their mean (±standard deviation) age was 21.3 (±1.7) years. The characteristics of the study subjects are shown in Table 1. The prevalence of stress of all levels was about 63.8%, and the prevalence of severe stress was 25.2% (Table 2).

The proportion of female students who had stress was higher (75.7%) than their counterpart males (57%) [χ^2^=27.2, odds ratio (OR)=2.3, p<0.0001].

The prevalence of stress was the highest among the first-year students (78.7%), followed by the second-year (70.8%), third-year (68%), fourth-year (43.2%), and fifth-year students (48.3%). There was a high significant association between the study year and the stress levels (χ^2^ test for trend=47.9, p<0.0001). The odds ratios were 3.96 (first year), 2.6 (second year), 1.9 (third year), and 0.82 (fourth year) respectively while the fifth year was considered the reference category. It also indicated a high significant association. The association between the academic grades of the students and the rate of stress was not significant (χ^2^ test for trend=1.01, p=0.31).

**Table 1.** Characteristics of study subjects

Study variable	No.	%
Gender (n=775)		
Male	495	63.9
Female	280	36.1
Year of study (n=774)		
1st	188	24.3
2nd	144	18.6
3rd	236	30.5
4th	148	19.1
5th	58	7.5
Grades (n=699)		
Excellent	357	51.1
Very good	191	27.3
Good	117	16.7
Poor	34	4.9
Regular in attendance (n=757)		
Yes	691	91.3
No	66	8.7

**Table 2.** Prevalence and levels of stress and physical problem

Variable	No.	%
Levels of stress (n=775)		
No stress	281	38.3
Mild	158	20.4
Moderate	141	18.2
Severe	195	25.2
Perception of physical problems (n=721)		
No	372	51.6
Mild to moderate	297	41.2
Severe	52	7.2

There was no significant association between the regularity of attendance in the academic course (yes/no) and the level of stress among the students. The distribution of stress levels was not significantly related to a student being either regular or irregular in attending the academic course work (χ^2^=0.037, OR=1.05, p=0.85). Students who reported physical symptoms were more likely to be stressed than those who did not perceive physical symptoms.

The presence of physical problems had a significant association with a higher level of stress among the students (χ^2^=20.3, OR=2.1; p<0.0001) (Table 3). The study variables, including being female (p<0.0001), year of study (p<0.001), and presence of perceived physical problems (p<0.0001), were found as independent significant risk factors for the outcome variable of stress (Table 4). The mean number of days (9.5 days) being unable to study or work was higher among students who had stress than students with no stress (2.3 days) (t=9.75, p<0.0001). The mean number of days that the respondents managed to work only partially was higher among students who had stress (10.7 days) than students with no stress (5.2 days) (t=5.3, p<0.0001).

**Table 3.** Association between stress and study variables (univariate analysis)

Study variable	Stress						
	Present		Absent		χ^2^ value	p value	OR (95% CI)
	No.	%	No.	%
Gender							
Male	282	56.9	213	43.1	27.2	<0.0001*	1.0
Female	212	75.7	68	24.3			2.3 (1.7-3.3)
Year of study							
1st	148	78.7	40	21.3	47.9	<0.0001*	3.96 (2.1-7.4)
2nd	102	70.8	42	29.2			2.60 (1.4-4.9)
3rd	151	64	85	36			1.90 (1.0-3.4)
4th	64	43.2	84	56.8			0.82 (0.44-1.5)
5th	28	48.3	30	51.7			1.0
Grade							
Excellent	236	66.1	121	33.9			1.2 (0.8-1.9)
Very good	120	62.8	71	37.2			1.05 (0.6-1.7)
Good	72	61.5	45	38.5	1.01	0.31	1.0
Poor	21	61.8	13	38.2			1.01 (0.4-2.2)
Regular in attendance							
Yes	442	64	249	36			1.0
No	43	65.2	23	34.8	0.037	0.85	1.05 (0.6-1.8)
Physical problems							
Yes	260	74.5	89	25.5	20.3	<0.0001*	2.1 (1.5-2.9)
No	217	58.3	155	41.7			1.0

*Significant;

CI=Confidence interval;

OR=Odds ratio

The main sources of stress stated by the students were coping with their studies (60.3%), followed by home environment (2.8%). However, 36.9% of the students did not mention any source of stress.

## DISCUSSION

A high prevalence of stress among medical students is a cause of concern as it may impair behaviour of students, diminish learning, and ultimately affect patient care after their graduation. The overall prevalence of stress in the study (63.7%) is similar to the Thai study (61.4%) (14) but higher than a study in Egypt (43.7%) (16), or a Malaysian study (41.9%) (13), and a British study (31.2%) (12). This could be either due to the different instruments used in other studies or it could be a real difference.

**Table 4.** Risk factors for stress (multivariate analysis)

Risk factor	Adjusted OR (95% CI)	p value
Gender (female)	2.7 (1.8-3.9)	<0.0001
Year of study		
1st	3.2 (1.6-6.2)	0.001
2nd	2.4 (1.2-4.8)	0.012
3rd	1.6 (0.87-3.1)	0.13
4th	0.45 (0.23-0.91)	0.02
Physical problem (yes)	2.01 (1.4-2.8)	<0.0001

OR=Odds ratio;

CI=Confidence interval

An interesting finding of the present study was that the level of stress decreased as the year of study progressed. This is contradictory to the finding of another study where the level of stress increased progressively during the course, to reach as high as 40% by the end of the clinical training period (24). Results of other studies in North America also suggest that mental health worsens after students join a medical school and remains poor throughout the course (25), especially in the transition from basic science teaching to clinical training (26). Only one study falls in line with the finding of this study that the students found medical course stressful during the first year of study but less so in subsequent years (27). This finding could be explained by many factors. First, that this is a cross-sectional and not a cohort study to be sure that the stress is really decreasing in the study subjects. This finding could be just due to chance as the study shows the increase of stress in different groups and not the same student groups. Another explanation could be that the students may have been able to develop coping mechanisms with the help of the students' support system. Also, usually low failure rate**s** in later years of courses make students more confident and less stressed. Another factor could be that our medical education is free of charge for the students in the governmental medical colleges. In many countries, medical students are plagued by financial worries, which is an important cause of their stress (7,28).

The present study did not show any association of stress with grade point average (academic grade) and regularity of attendance in the course**s**. However, stress was significantly associated with the students' perception of physical problems. This aspect is difficult to explain based on the findings of the study. It is possible that stress is the cause of the physical symptoms, or the physical symptoms could cause the stress—both are possible.

The prevalence of stress in the study was higher among the female students compared to their male counterparts but other studies have shown that the gender differences in specific stress symptoms and overall prevalence or mean scores of stress were scarce and did not turn out to be a significant factor in reporting of stress (12,23,24). As male and female students in their studies have separate campuses, it could be speculated that relatively a poor learning environment exists in the female campus with lesser educational facilities and recreation opportunities. However, this issue could not appropriately be explained by our study and requires further investigation.

The negative effects of long and tiring medical education on the psychological status of students have been shown in several studies. Results of a study in the UK showed that one-third of psychologically-ill students did not graduate from the college (29). The changes relating to becoming a medical student appear to have a significant impact on the psychological status of students during the first year in their study. Therefore, with early identification and effective psychological services, possible future illnesses may be prevented. As the study findings showed a high level of stress among the first-year and second-year students, we suggest supporting them and taking care of this group by the student support system. This will also help them cope well with stress in the later years. It is very important to target stress-prevention strategies at students who have any level of psychological stress to prevent the development of more serious conditions relating to stress. Wellness and mental health programmes are also needed to help students make smooth transition between different learning environments with changing learning demands and a growing burden on their mental and physical capacity. Medical schools in the United States and Canada have initiated health-promotion programmes and have reported positive results in reducing the negative effects of stress upon health and academic performance of medical students (30-32). A similar approach to reduce the level of stress could be used for the students of the College of Medicine, King Saud University. On the other hand, a minimal amount of stress is necessary to add spice to one's life and to achieve optimal performance at examinations. An element of stress is involved with growth and is essential for sound personal functioning.

### Limitations

This cross-sectional study was based on self-reported information provided by students. Therefore, there is some potential for reporting bias which may have occurred because of the respondents' interpretation of the questions or desire to report their emotions in a certain way or simply because of inaccuracies of responses. Another longitudinal study could be carried out with a cohort of students to investigate the levels of stress among students in all the five years of undergraduate medical years and the associated factors.

### Conclusions

The findings of the study suggest that the level of psychosocial stress was higher in the female students compared to the male students. The stress level in the initial three years of the course was higher than the last two years of the course. Physical problems might have led to extra stress. The study did not find a significant association between academic grades and regularity of attendance in the course on one hand and the presence of stress on the other hand. The findings of high level of stress among the medical students in the initial years also suggest that, when students are admitted to the medical school, special care must be taken to find out obvious psychiatric problems or psychological stress among them. The major finding of high psychological stress in the students of the medical college of King Saud University points to the need for establishing counselling and preventive mental health services as an integral part of routine clinical services being provided to the medical students.

## ACKNOWLEDGEMENTS

The authors gratefully acknowledge all those medical students who participated in and contributed to the study. They also thank Dr. Shaffi Ahamed Shaikh for statistical analysis and Prof. Riaz Qureshi for revising and editing the manuscript.
